# Chemokine Binding Protein M3 of Murine Gammaherpesvirus 68 Modulates the Host Response to Infection in a Natural Host

**DOI:** 10.1371/journal.ppat.1001321

**Published:** 2011-03-17

**Authors:** David J. Hughes, Anja Kipar, Gail H. Leeming, Elaine Bennett, Deborah Howarth, Joanne A. Cummerson, Rita Papoula-Pereira, Brian F. Flanagan, Jeffery T. Sample, James P. Stewart

**Affiliations:** 1 School of Infection and Host Defence, The University of Liverpool, Liverpool, United Kingdom; 2 Department of Microbiology and Immunology, Pennsylvania State University College of Medicine, Hershey, Pennsylvania, United States of America; 3 Veterinary Pathology, School of Veterinary Science, The University of Liverpool, Liverpool, United Kingdom; Oregon Health & Science University, United States of America

## Abstract

Murine γ-herpesvirus 68 (MHV-68) infection of *Mus musculus*-derived strains of mice is an attractive model of γ-herpesvirus infection. Surprisingly, however, ablation of expression of MHV-68 M3, a secreted protein with broad chemokine-binding properties *in vitro*, has no discernable effect during experimental infection via the respiratory tract. Here we demonstrate that M3 indeed contributes significantly to MHV-68 infection, but only in the context of a natural host, the wood mouse (*Apodemus sylvaticus*). Specifically, M3 was essential for two features unique to the wood mouse: virus-dependent inducible bronchus-associated lymphoid tissue (iBALT) in the lung and highly organized secondary follicles in the spleen, both predominant sites of latency in these organs. Consequently, lack of M3 resulted in substantially reduced latency in the spleen and lung. In the absence of M3, splenic germinal centers appeared as previously described for MHV-68-infected laboratory strains of mice, further evidence that M3 is not fully functional in the established model host. Finally, analyses of M3's influence on chemokine and cytokine levels within the lungs of infected wood mice were consistent with the known chemokine-binding profile of M3, and revealed additional influences that provide further insight into its role in MHV-68 biology.

## Author Summary

Infection of inbred strains of laboratory mice (*Mus musculus*) with the rodent γ-herpesvirus MHV-68 continues to be developed as an attractive experimental model of γ-herpesvirus infection. In this regard, the MHV-68 protein M3 has been shown to selectively bind and inhibit chemokines involved in the antiviral immune response, a property expected to contribute significantly to virus infection and host colonization. However, inactivation of the *M3* gene has no discernable consequence on infection in this animal host. Prompted by recent evidence that natural hosts of MHV-68 are members of the genus *Apodemus,* and that MHV-68 infection in laboratory-bred wood mice (*Apodemus sylvaticus*) differs significantly from that which has been described in standard strains of laboratory mice, we addressed whether M3 functions in a host-specific manner. Indeed, we find that M3 is responsible for host-specific differences observed for MHV-68 infection, that its influence on infection within wood mice is consistent with its chemokine-binding properties, and that in its absence, persistent latent infection - a hallmark of herpesvirus infections - is attenuated. This highlights the importance of host selection when investigating specific roles of pathogenesis-related viral genes, and advances our understanding of this model and its potential application to human γ-herpesvirus infections.

## Introduction

The human γ-herpesviruses - Epstein-Barr virus (EBV) and Kaposi's sarcoma-associated herpesvirus (KSHV; alternatively human herpesvirus 8 [HHV-8]) - possess significant oncogenic potential, particularly in the setting of immune deficiency. Both establish lifelong latent infections, primarily within B lymphocytes, through the actions of a limited repertoire of their approximately 90 genes. While the majority of these have a role in virus production, it is principally the actions of the latency-associated genes of these viruses that contribute to their oncogenic potential. Strict host preferences of EBV and KSHV, unfortunately, severely limit assessment of the mechanisms that contribute to their persistence and pathogenesis. Consequently, there has been considerable effort to develop experimental infection of laboratory mice (*Mus musculus*) with the murine γ-herpesvirus 68 (MHV-68 or γHV68; officially murid herpesvirus 4 [MuHV-4]) as a model of γ-herpesvirus infection [1], [2], [3], [4], [5], [6], [7].

As a member of the γ_2_ subfamily of herpesviruses, MHV-68 is closer genetically to KSHV/HHV-8 than to EBV, a γ_1_ herpesvirus [8], [9]. Regardless, each γ-herpesvirus contains a unique set of genes that contributes to its distinct biology and pathogenic properties. For MHV-68, this is primarily a cluster of latent- and lytic-infection-associated genes at the extreme left end of the viral genome that encodes for four novel proteins, M1–M4, and interspersed among these are eight RNA polymerase III-transcribed genes that encode abundant viral *tRNA*-like (*vtRNA*) transcripts [8], [10]. Much of the effort to define the biology of MHV-68 infection and its applicability as a model of human γ-herpesvirus infections, has therefore focused on the roles of these genes in the context of infection within inbred strains of laboratory mice. Of the proteins encoded by this locus, the biochemical function of M3 is the best understood.

A secreted 44-kDa protein, M3 is highly expressed during lytic infection, and probably to a lesser extent during latency [11], [12], [13], [14]. *In vitro,* M3 selectively binds chemokines associated with the antiviral inflammatory response [15], [16]. Surprisingly, inactivation of M3 expression (by insertion of a translational stop codon) has no apparent consequence on MHV-68 infection following intranasal inoculation of C57BL/6 mice [17]. By contrast, intracerebral injection of the same *M3* mutant virus does lead to an altered inflammatory response, with higher numbers of infiltrating lymphocytes and macrophages than observed following inoculation with wild-type virus [17]. Thus, M3 does appear capable of functioning as a chemokine-binding protein *in vivo*, though it is perplexing why ablation of M3 expression has no apparent impact on pathogenesis or on virus replication and the establishment of latency following intranasal inoculation, clearly more representative of a natural route of infection. One possible explanation for this may relate to the experimental host.

MHV-68 was originally isolated from a bank vole (*Myodes glareolus*) [18] although this appears to be only an occasional host [19]. In spite of what has been suggested recently [20], we have shown conclusively using sequence analysis that the natural hosts of MHV-68, at least in continental Europe, are in fact members of the genus *Apodemus* [21]. Specifically, *Apodemus flavicollis*, *Apodemus agrarius*, and *Apodemus sylvaticus* (wood mice) [21]. Significantly, our recent comparative analysis of experimental MHV-68 infection of BALB/c (*M. musculus*) and laboratory-bred wood mice revealed markedly different findings [22]. In wood mice, virus replication in the lung was substantially muted, and latency within the spleen was established without the dramatic leukocytosis and splenomegaly that are the hallmark pathogenic properties of MHV-68 latency within inbred laboratory strains of mice. In addition, the associated histological changes were significantly different. Notably, in wood mice, viral replication was restricted to scattered alveolar epithelial cells and macrophages within focal granulomatous infiltrations. Latently-infected lymphocytes were also abundant in focal perivascular/peribronchiolar infiltrations and in inducible bronchus-associated lymphoid tissue (iBALT). In addition, while well-delineated secondary follicles with classical germinal center formation were seen in the wood mouse spleens, only poorly-delineated follicles without distinct germinal centers were seen in BALB/c mice.

Given the unlikelihood of an insignificant role for M3, we asked whether M3 might contribute to the vastly different response of wood mice to MHV-68 infection. Here we demonstrate that upon intranasal inoculation of wood mice, M3 does indeed modulate the host inflammatory response in a manner consistent with its chemokine-binding properties, and that it is responsible for the MHV-68-dependent iBALT observed in this species. Additionally, we show that M3 is critical for the organization of splenic follicles, and that in the absence of M3, latent MHV-68 infection is significantly attenuated in both lung and spleen. These results highlight the importance of utilizing a natural host in this small-animal model of γ-herpesvirus infection, and provide substantial new insight into the biology of MHV-68 that should contribute to future use of this model and its applicability to understanding human γ-herpesvirus infections and pathogenesis.

## Results

### Expression of *M3* during acute infection of wood mice

Following intranasal inoculation of mice, e.g., BALB/c and C57BL/6, a burst of MHV-68 replication occurs within lung epithelial cells [23] prior to the establishment of latent infection within lung epithelium [24] and ultimately the hematopoetic system [25], [26]. This replication peaks at approximately 7 days p.i. and is largely resolved by day 10 p.i.. The titer of virus produced in the lungs of wood mice, however, is substantially lower (by ∼3 log_10_ plaque forming units), though the long-term viral DNA loads established within the lung of wood and BALB/c mice are equivalent [22]. In contrast to BALB/c mice, virus productive replication in the lungs of wood mice appears confined within granulomatous infiltrates, and in separate lesions, numerous lymphocytes within perivascular and peribronchial accumulations harbor latent virus (primarily within B cells). We reasoned, therefore, that the immune-modulatory function of M3 might be particularly critical during acute infection within the lungs of wood mice. To address this, we first examined *M3* expression in lung by quantitative reverse transcription PCR (qRT-PCR). To put our results in a more meaningful perspective, we determined *M3* mRNA levels relative to those for the other 3 genes in this locus (*M1*, *M2* and *M4*), as well as to the early lytic-cycle gene *ORF50*, which served as a general indicator of lytic infection. This experiment was performed twice with comparable results.

**Figure 1:**
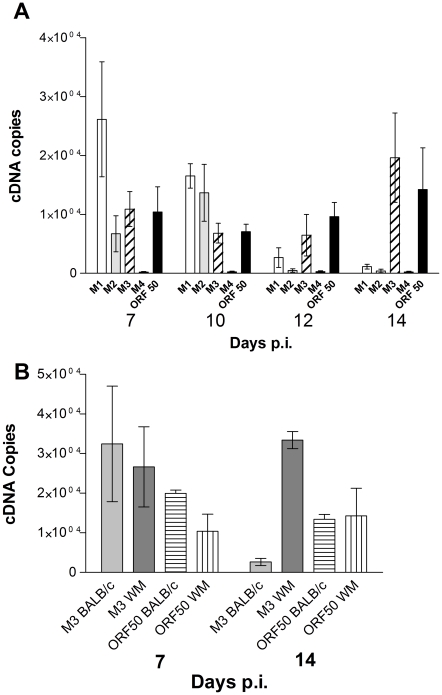
Comparative analysis of *M3* RNA expression in lungs of infected wood mice. Quantification by qRT-PCR of RNA expressed from the MHV-68 genome within lungs; *ORF50* RNA levels were assessed as a general reference for lytic-cycle gene expression. The copy numbers of individual viral-gene RNAs (as cDNAs) were normalized to those for cellular *RPL8*. Error bars represent the standard error of the mean from three wood mice per time point. (A) analysis of expression from the *M1-M4* locus of infected wood mice lungs. (B) analysis of M3 expression from the lungs of BALB/c and wood mice. Note that, although *M3* expression is similar for both species at 7 days p.i., after 14 days p.i., *M3* expression in BALB/c mouse lungs is drastically reduced compared to wood mice.

As illustrated in Fig. 1A, *M* *3* expression was detected at all four time points evaluated (7, 10, 12, and 14 days p.i.). There were, however, two unexpected findings with respect to *M3*. First, whereas *M3* encodes one of the most highly expressed MHV-68 mRNAs during lytic infection *in vitro*, especially relative to the other lytic-cycle mRNAs encoded by this locus (*M1* and *M4*) [27], this was clearly not the case *in vivo* here. Second, there was an obvious spike in *M3* expression between 12 and 14 days p.i. (Fig. 1A), a time when virus replication is believed to have subsided. Clearly, the level of *M3* expression at 14 days p.i. was significantly higher than that for *M1*, *M2* and *M4* (*P*<0.01). Although this may be indicative of the onset of latency-associated *M3* expression, detection of a parallel spike in *ORF50* expression suggests that this is lytic cycle-associated expression. However, we cannot exclude the possibility that at this point in infection, *M3* transcripts originate from latently infected cells, whereas *ORF50* expression is occurring in separate cells still supporting full or an abortive virus replication, probably within granulomatous infiltrates that support productive infection in wood mice lungs [22].

**Figure 2:**
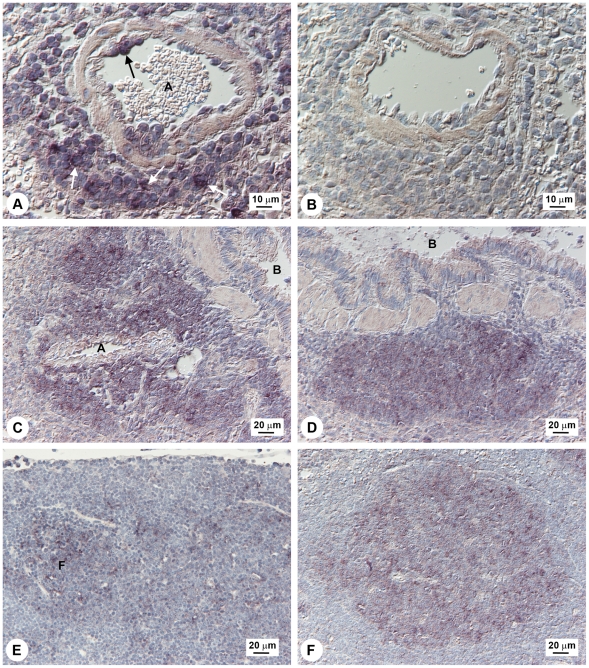
Localization of *M3* expression in lung, spleen, and lymph node. Detection of *M3* RNA in lung and spleen by *in situ* hybridization in MHV-68 infected wood mice. A, B: Lung, day 7 p.i.: (A) *M3* transcripts detected within lymphocytes in perivascular infiltrates (white arrows) and lymphocytes attached to the endothelial wall (black arrow). A: artery; (B) No signal detected with sense-strand probe (negative control). C, D: Lung, day 12 p.i.: (C) Perivascular and peribronchial lymphocyte infiltrations containing numerous *M3*-positive lymphocytes. A, artery; B, bronchioles; (D) Peribronchial, focal follicle-like lymphocyte accumulation with numerous *M3*-positive lymphocytes. B, bronchiolus. (E) Bronchial lymph node, day 7 p.i.; lymphocytes showing *M3* transcripts are present in lymphatic follicles (within germinal center cells). F, follicle. (F) Spleen, day 14 p.i.; follicle with numerous *M3* RNA-positive lymphocytes in the germinal center. Results are representative of numerous tissue sections analyzed from three infected wood mice.

There were several additional observations of note with respect to this gene locus. Whereas *M1* mRNAs are relatively low in abundance during MHV-68 replication *in vitro* and in BALB/c mice spleens [28], *M1* expression was significantly higher (*P*<0.05) at 7 days p.i. than the other genes within the locus, and still one of the most highly expressed genes tested at 10 days p.i. (Fig. 1A). By contrast, *M4* transcript levels were nominal, suggesting that M4 either performs a function at times or anatomical sites other than those analyzed here, or that a comparatively lower level of *M4* transcript is required for M4 expression. Finally, it was somewhat surprising that expression of *M2*, believed to be a strict latency-associated gene [29], was readily detectable early in the infection, peaked at 10 days p.i. and decreased through day 14 p.i. where numerous latently-infected B cells are known to be present [22]. MHV-68 latency has been detected as early as 3 days p.i. [30] and B cell infiltrations containing MHV-68 are present as early as day 7 p.i. in the wood mouse (Fig. 2), [22]. Further, it has been shown that the pattern of MHV-68 latent gene expression is differentially regulated in B cells depending on cellular differentiation state [31] and thus the observed pattern of M2 expression is likely a reflection of this.

To assess the relative level and timing of M3 expression between wood and BALB/c mice we determined *M3* and *ORF50* mRNA levels in the lungs of infected wood and BALB/c mice at 7 and 14 days p.i. by qRT-PCR as above. The results (Fig. 1B) showed that *M3* expression in BALB/c mice was similar to wood mice at day 7, but significantly lower (*P*<0.01) at day 14, showing that the timing of *M3* expression differs between the two species of host.

We next sought to localize the site of *M3* transcription within lung and spleen by RNA *in situ* hybridization. Within lung, at 7 days p.i. *M3-*positive lymphocytes were detected in B cell-dominated perivascular/peribronchial infiltrates and, together with positive macrophages, within granulomatous infiltrates and occasionally within blood vessels, rolling along/attached to vascular endothelial cells (Fig. 2A). This *M3* RNA expression pattern was seen also on days 10, 12 and 14. On days 12 and 14 p.i., there were many *M3* positive lymphocytes in the progressively prominent perivascular/peribronchial lymphocyte accumulations (Fig. 2C), and some were also seen disseminated in the parenchyma. They were also present in the follicle-like B-cell accumulations that were first seen on day 12 (Fig. 2D) and which had developed germinal centers by day 14 (data not shown). Previous work has shown that these follicle-like infiltrations with germinal centers are inducible bronchus-associated lymphoid tissue (iBALT). Consistent with a trafficking of latently infected cells from the site of primary replication within the respiratory tract to spleen, we observed variable numbers of *M3*-positive lymphocytes mainly in follicles within bronchial and mandibular lymph nodes on day 7 p.i. (Fig. 2E). Within spleen, in which latently infected cells peak approximately 12–16 days p.i., *M3* expression was prominent within follicle centers from 10 days p.i. onward (Fig. 2F). No hybridization was detected in any tissue with sense-strand probes (e.g., Fig. 2B).

Thus, a high level of *M3* expression was observed at d14 p.i. in lymphocytes within iBALT and splenic follicles.

### Loss of M3 alters the host response to MHV-68 infection in lung

**Figure 3:**
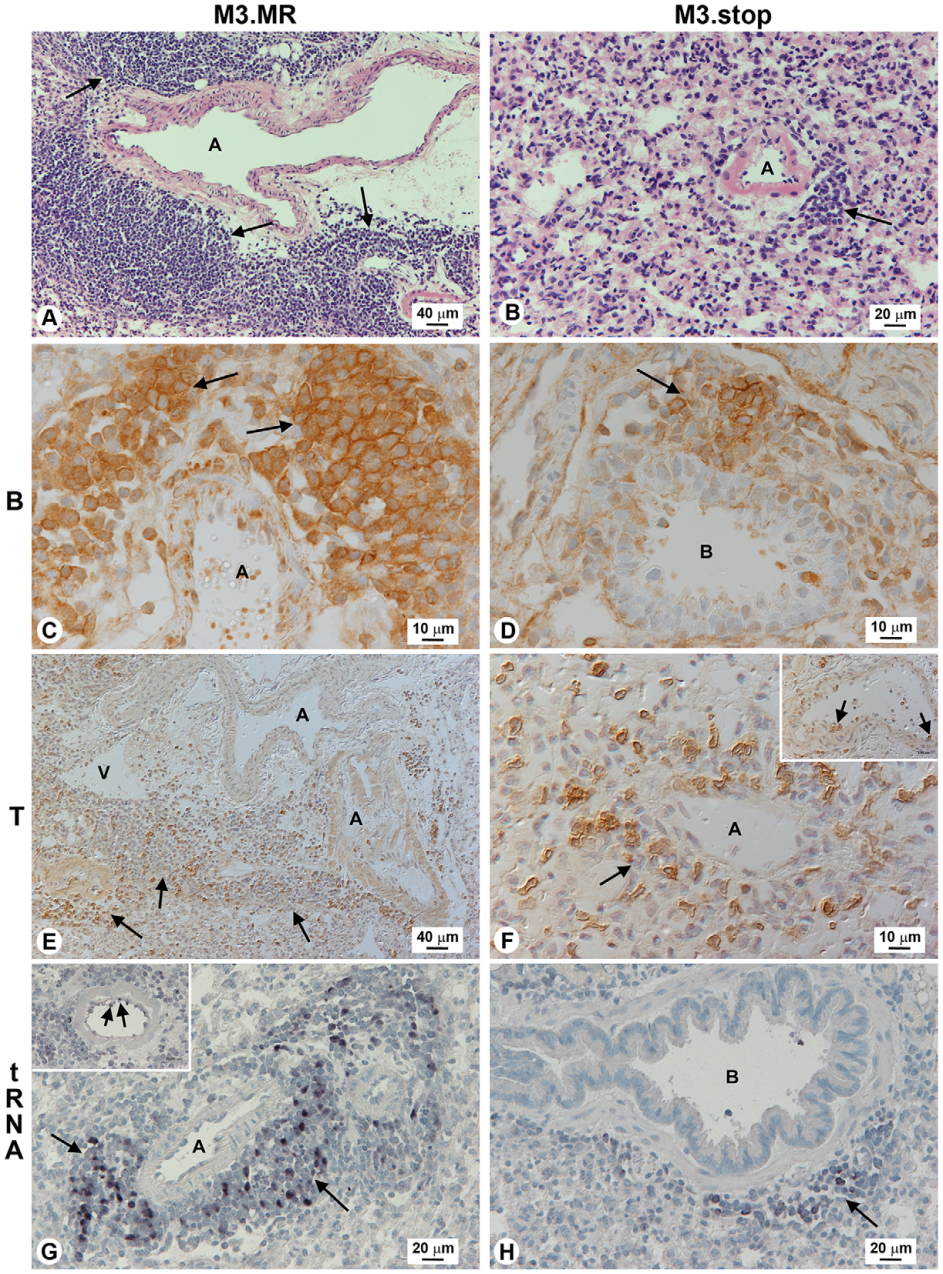
Influence of M3 in lung of acutely infected wood mice. All data were from lungs of mice infected with either M3.MR (left panels) or M3.stop virus (right panels) at 14 days p.i. (A) M3.MR (i.e., pseudo wild-type MHV-68) infected wood mouse lung tissue (HE stain) showing large perivascular lymphocyte infiltrations; arrows indicate iBALT. (B) M3.stop infected wood mouse lung showing much smaller perivascular lymphocyte infiltration (arrow). Panels (C) and (D): Immunohistological staining showing B-cell (CD45R^+^) dominance in the peribronchial infiltrations (iBALT) of mice infected with M3.MR (C; arrows), and the reduced proportion of B cells in the equivalent infiltrates in mice infected with M3.stop virus (D; arrow). Panels (E) and (F): Immunohistological staining for T cells (CD3^+^) indicating that they are a minority cell type in iBALT within M3.MR-infected mice (E; arrows), whereas T cells are more prevalent in the infiltrates within M3.stop-infected mice (F; arrow); T cells were also seen rolling along endothelial walls (F; inset and arrows). Panels (G) and (H): Detection by *in situ* hybridization of *vtRNA* expression (indicative of latent infection). In mice infected with M3.MR, positive cells are numerous in the perivascular infiltrates (G; arrows) and are seen attached to the endothelial wall (G; inset and arrows). They are present but less numerous in mice infected with M3.stop (H; arrow). All images are representative of numerous tissue sections analyzed from 3 wood mice per infection. Labels: A, artery; V, vein; B, bronchiole.

**Figure 4:**
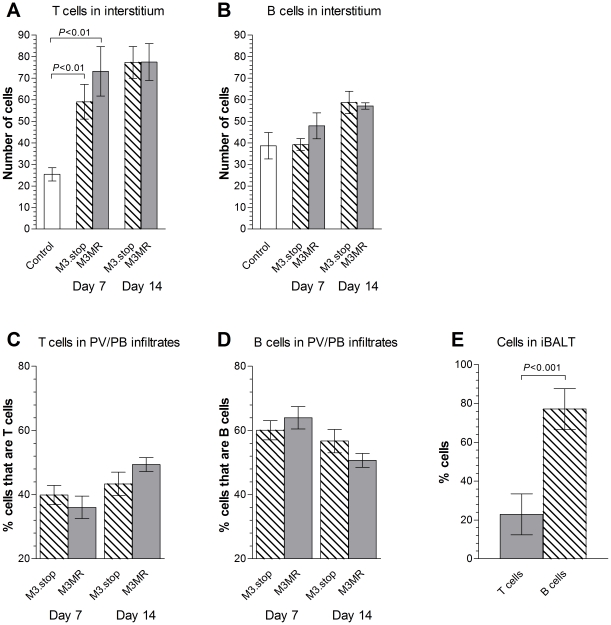
Influence of M3 on T and B cell numbers in the lungs of acutely infected wood mice. All data were from mice infected with either M3.MR or M3.stop virus at either 7 or 14 days p.i. as indicated. Quantification of B and T cell infiltrates was performed by counting the number of cells in corresponding sequential sections from the same histopathological sections as in Fig. 3. The number of cells per unit area (138138 µm^2^) was then calculated for the interstitial infiltrate (Panels A and B) and the proportion of B and T cells for each area of perivascular/peribronchiolar (PV/PB) infiltration (Panels C, D) and iBALT (Panel E). Data are shown as the mean values ± SEM and compared between groups using a two sample *t*-test.

We next asked if the development of B cell-dominated, *M3* RNA-positive perivascular/peribronchial infiltrates (Fig. 2A) and iBALT, which are not features of the lungs of BALB/c mice infected with MHV-68, was a result of M3 expression. To address this question, we infected cohorts of wood mice with a previously characterized recombinant MHV-68 that has a targeted disruption of the *M3* gene (M3.stop, gift of S.H. Speck and H.W. Virgin) [17]. The *M3* gene in this virus contains three translational stop codons inserted into the 5′ end of the *M3* ORF. The marker-rescue version of this virus, M3.MR, containing a fully restored *M3* gene [17], was used as wild-type virus for comparison. The histopathological analyses are shown in Fig. 3 and the quantification of these in Fig. 4). As expected, the histological changes in the lung tissue from wood mice infected with M3.MR on day 7 and 12 p.i. were similar to those observed with MHV-68 infection as follows [22]. There was a marked increase in the amount of interstitial lymphocytes based on the significant (*P*<0.01), ca 3 fold increase in T cells as compared with uninfected animals (Fig. 4A, B). There was also a moderate perivascular and peribronchial infiltration which contained a higher proportion of B cells (B∶T ratio of 1.77∶1, *P*<0.001; Fig. 4C, D) with B cell rolling and emigration (data not shown). Multifocal granulomatous infiltrates containing viral antigen were also observed. By 14 days p.i., two types of lymphocyte-dominated perivascular and peribronchial infiltrations had developed multifocally in association with larger arteries and bronchi. One type contained T and B cells in approximately equal proportions (Fig. 4C, D), and the other was B-cell dominated and follicle-like with germinal center formation, i.e., iBALT (Fig. 3A, C; 4E). B lymphocytes made up ca 75% of the cells in iBALT (*P*<0.001), whereas T cells were present in much smaller numbers (Fig. 3C,E; 4E). While evidence of iBALT formation was already seen on day 12 p.i., the perivascular/peribronchial infiltrates that contained approximately equal proportions of T and B cells were only seen after 14 days p.i. and the latter perhaps represent the physiologic immune response as the acute phase of MHV-68 infection in lung dwindles. Lymphocytes that expressed *vtRNA*, indicative of a latent infection, were found within iBALT, and very occasionally intravascularly (Fig. 3G).

By contrast, infection with M3.stop virus led to markedly different histological findings. At 7 days p.i., a statistically-significant (*P*<0.01) increase in interstitial lymphocytes that consisted of predominantly T cells was observed (Fig. 4A, B). Multifocal granulomatous infiltrates containing viral antigen were also observed. Mild perivascular/peribronchial lymphocyte accumulations were obvious, and immunohistological staining showed that this was B-cell dominated (B∶T ratio of 1.5∶1, *P*<0.001; Fig. 4C, D). T cells were also seen rolling along and emigrating from blood vessels, an observation not seen when *M3* was expressed. After 14 days, the perivascular and peribronchial lymphocyte infiltrations were still evident (Fig. 3B). However, these were far less intense than in the lungs of the M3.MR-infected wood mice (compare to Fig. 3A) and consisted of both B cells (Fig. 3D) and T cells (Fig. 3F) in a ratio of 1.3∶1 (Fig. 4C, D). T cells (but not B cells) were also found rolling along arterial walls and emigrating from vessels of M3.stop-infected animals (Fig. 3F, inset). *vtRNA*-positive lymphocytes were observed in perivascular infiltrates of M3.stop-infected wood mice, but there were fewer of these, possibly due to the lower proportion of B cells and the smaller size of the infiltrates (Fig. 3H) than seen for infection with M3.MR virus (compare to Fig. 3G). Notably, while granulomatous infiltrates were seen in both groups of mice, iBALT was absent in M3.stop-infected mice. Thus, while M3 is not essential for infection, the host response to infection is clearly altered in its absence.

### Lack of M3 alters the germinal center reaction in spleen

**Figure 5:**
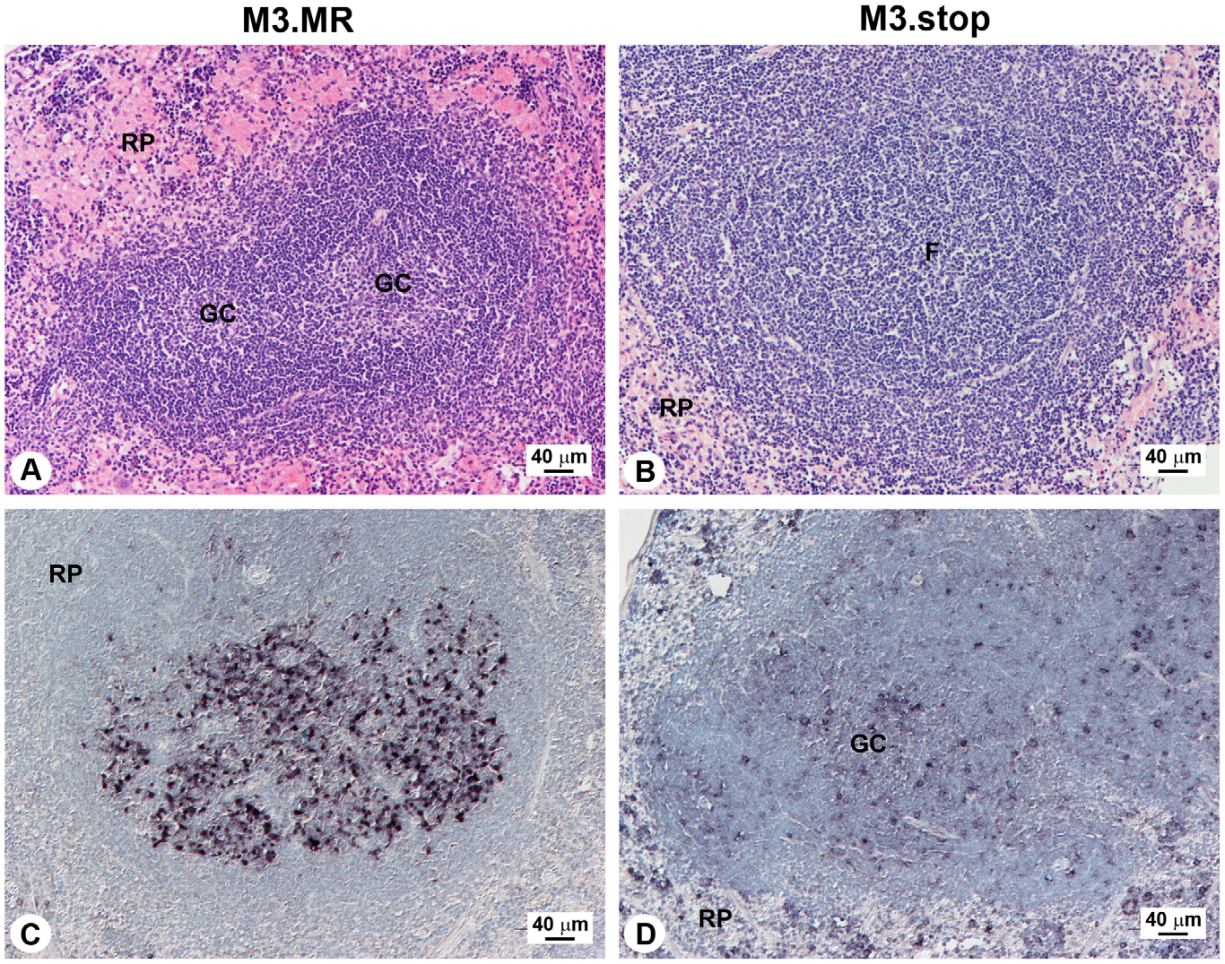
Influence of M3 in spleen of acutely infected wood mice. All data were from mice infected with either M3.MR or M3.stop virus at 14 days p.i. (A) In M3.MR infected mice, the white pulp is composed of secondary follicles with distinct germinal centers, exhibiting obvious light and dark zones. HE stain. (B) In M3.stop-infected mice, follicles are larger and exhibit large, poorly delineated germinal centers. HE stain. (C) Localization, by *in situ* hybridization to *vtRNAs*, of latently infected splenocytes within spleen of M3.MR-infected mice. Latently infected cells are primarily contained within the light zone of germinal centers. (D) *vtRNA* detection within latently infected splenocytes in M3.stop-infected mice. Note that positive cells are found scattered throughout the follicle, as well as in the red pulp. Labels: F, follicle; GC, germinal center; RP, red pulp.

A major organ of MHV-68 persistence is the spleen, in which the number of latently infected cells - primarily B cells but also dendritic cells and macrophages - peaks approximately 2 weeks p.i. [5], [32]. As *M3* is expressed within spleen (Fig. 2F), we examined the effect that M3 loss has on MHV-68 infection there. As shown in Fig. 5A, at 14 days p.i. the spleens of wood mice infected with M3.MR virus contained moderately sized follicles with distinct germinal centers. By contrast, the spleens of M3.stop-infected animals displayed expanded follicles without distinct germinal centers, and a slight increase in cellularity of the red pulp (Fig. 5B). Interestingly, splenic architecture observed in mice infected with M3-stop virus was very similar to that observed in the spleens of BALB/c mice infected with MHV-68, but without the marked increase in the number of leukocytes within the red pulp [22]. Further, identification of *vtRNA*-positive cells by *in situ* hybridization indicated that the well-delineated splenic follicles of M3.MR-infected mice were heavily populated with latently infected cells, and that these cells were rare outside follicles (Fig. 5C), comparable to what we had observed upon infection with wild-type MHV-68 [22]. Although *vtRNA*-positive cells were detected within the poorly-defined follicles of M3.stop-infected mice, the number of these cells was notably lower, and they were occasionally present as well within the red pulp (Fig. 5D). However, consistent with the inability of MHV-68 to induce significant leukocytosis and splenomegaly in wood mice (unlike in BALB/c and C57BL/6 mice), we noted no significant change in total spleen cell numbers after infection with either M3.MR or M3.stop (data not shown). Thus, the *M3* gene clearly influences MHV-68 infection within the spleen of wood mice and upon its inactivation, splenic architecture resembles that in BALB/c mice infected with wild-type MHV-68.

### M3 is required for efficient establishment of latency

**Figure 6:**
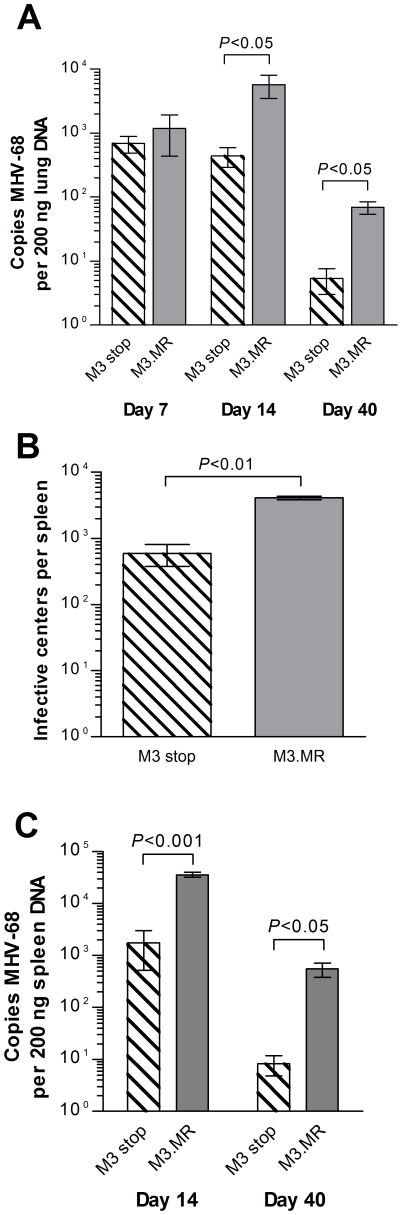
Contribution of M3 to MHV-68 infection in wood mice. (A) Loss of M3 is associated with reduction of viral DNA in lung at 7, 14 and 40 days p.i. Quantitative PCR analysis of viral genome copies per 200 ng lung DNA. (B) Reduction in latently infected cells in the spleen at 14 days p.i. as a consequence of M3 loss, as determined by infective center assay. (C) Reduction in viral DNA in spleen at 14 and 40 days p.i. parallels the loss of latently infected splenocytes in (B). Quantitative PCR analysis of viral genome copies per 200 ng spleen DNA. Data in (A) and (C) represent the mean value determined from three individual wood mice per infection, normalized to copies of cellular *rpl8* per DNA sample; error bars depict the standard error of the means. Statistical analysis was performed using Student's *t*-test and where significance was found this is indicated above the bars.

Given the dramatic histological differences that we observed in the lung and spleen as a consequence of disrupting M3 expression, we next asked how inactivation of M3 expression affected MHV-68 infection itself within these organs. Because MHV-68 replication within the lungs of wood mice does not yield the high titers of virus seen in BALB/c mice that can be readily quantified by plaque assay [22], we chose to indirectly measure levels of virus by qPCR. At 7 days p.i., the level of viral DNA detected within the lungs of mice infected with M3.MR was not significantly higher than that within the lungs of mice infected with M3.stop (Fig. 6A). At day 14, however, significantly reduced levels of viral DNA (*P*<0.05) were detected in the lungs of M3.stop-infected wood mice (Fig. 6A). A similar result was seen at day 40 p.i. when MHV-68 DNA was detected at low levels in M3.MR-infected mice, but at a significantly lower level in those infected with M3.stop (*P*<0.05).

To assess the effect of M3 loss in the spleen, infective center assays were performed at 14 days p.i. to measure the number of latently infected cells, which are normally at their peak level at this time. Similar to our observation in the lungs, the number of spleen cells that harbored reactivatable virus was significantly lower (*P*<0.005), though still detectable, in wood mice that had been infected with M3.stop (Fig. 6B). A parallel effect was seen when viral DNA was measured by qPCR (Fig. 6C), confirming that the disparity in infective centers was not due to an inability of M3.stop virus to reactivate *ex vivo*. Viral DNA was still detectable in both groups of wood mice at day 40 p.i., but at a significantly reduced level in the animals infected with M3.stop (Fig. 6C). Hence, in both lung and spleen, the lack of M3 significantly reduced the ability of M3.stop to establish a normal level of infection that, at least in spleen, reflected a nearly ten-fold lower number of latently infected cells.

### M3 modulates the pulmonary chemokine and cytokine response

**Figure 7:**
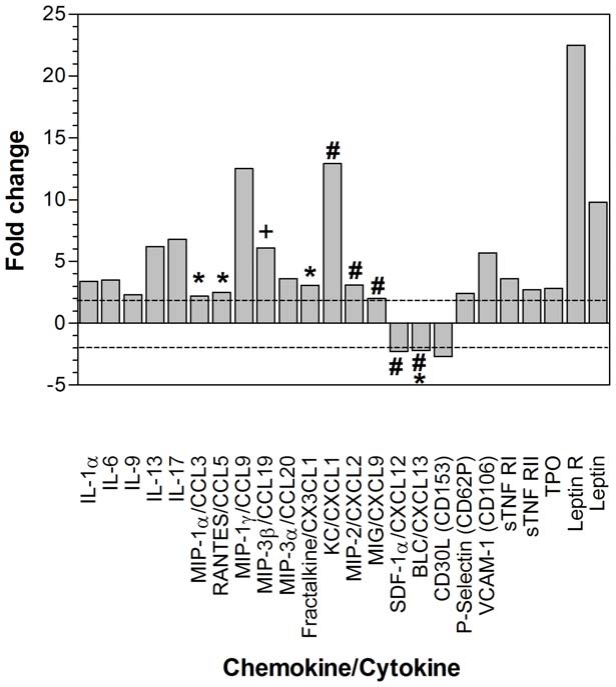
Array analysis of changes in pulmonary chemokine and cytokine levels within infected wood mice as a consequence of M3 loss. Equal amounts of protein from lung lysates of wood mice infected with M3.stop or M3.MR MHV-68 were incubated with RayBiotech 3.1 membrane arrays capable of detecting 61 different chemokines and cytokines. Shown are the relative abundance of cytokines and chemokines (minus background) whose expression consistently showed more than a two-fold difference in M3.stop- versus M3.MR-infected mouse lung lysate (two independent experiments). Asterisks denote chemokines that have been tested *in vitro* and found to be bound by M3; # denotes chemokines tested and not bound by M3, according to van Berkel *et al.* [16] and Parry *et al*. [15]; + denotes that MIP-3/CCL19-dependent chemotaxis is inhibited by M3 according to Jensen *et al.* [46]; #* denotes that in the case of BLC/CXCL13, while Parry *et al*. [15] and Martin *at al*. [62] found that M3 bound weakly and inhibited factor-dependent chemotaxis, van Berkel *et al.* [16] did not observe any binding to M3.

Because M3 is not required for replication of MHV-68 *in vitro* [17], [33], we reasoned that deficiencies of the M3.stop virus apparent in wood mice were more likely due to a loss of the chemokine-binding properties of M3, rather than to a direct defect in virus replication *per se*. To determine if loss of M3 expression results in a change in the chemokine profile, we measured the relative levels of a panel of chemokines and cytokines within the lungs of mice at 14 days p.i. (the peak of *M3* expression during acute infection; Fig. 1A) with either M3.MR or M3.stop virus. To accomplish this we performed cytokine antibody array analyses (RayBio Mouse Cytokine Antibody Array 3.1), a proven method of comparing cytokine/chemokine levels in tissues [34]. The results (Fig. 7) showed that in a number of cases, the amount of these molecules was notably higher (>2 fold positive fold change) in the lungs of mice infected with M3.stop relative to M3.MR virus. Specifically, we observed relative increases in RANTES/CCL5 (2.5-fold), MIP-1α/CCL3 (2.2-fold), fractalkine/CX3CL1(3-fold) KC/CXCL1 (12.9-fold), MIP-2/CXCL2 (3.1-fold) and MIG/CXCL9 (2.0-fold) in the absence of M3. By contrast, we observed relative decreases in the B-cell associated chemokines BLC/CXCL13 (2.2-fold) and SDF-1α/CXCL12 (2.3-fold), as well as CD30L (2.7-fold), in infections lacking M3 (Fig. 7).

**Figure 8:**
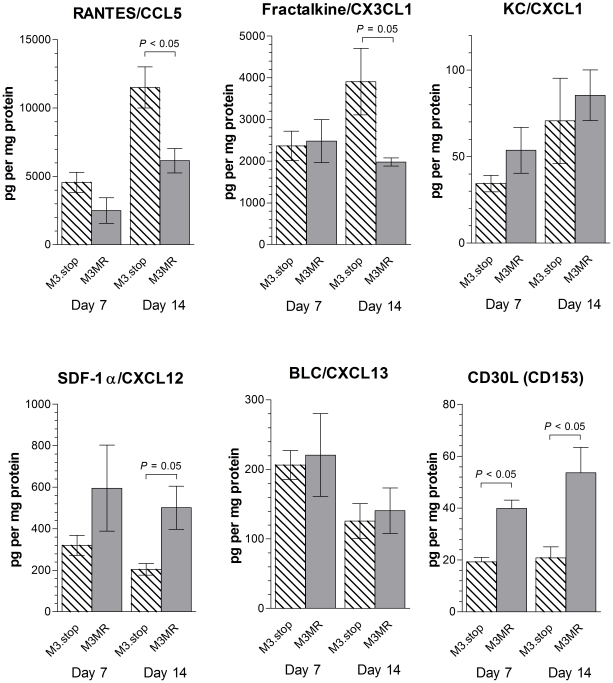
Influence of M3 on the changes in chemokine levels in the lungs of infected wood mice. Protein from lung lysates of wood mice infected with M3.stop or M3.MR MHV-68 were analysed for the concentration of specific chemokines by ELISA. Data were normalized relative to total protein concentration as determined by a modified Bradford assay. Error bars depict the standard error of the means for 4 individual wood mice. Statistical analysis was performed using Student's *t*-test and where significance was found this is indicated above the bars.

To confirm the above array results, the concentrations of selected chemokines were measured in the lungs of infected mice at 7 and 14 days p.i. by ELISA. The results (Fig. 8) showed that, in agreement with the array results, at day 14 p.i. the concentrations of RANTES/CCL5 and fractalkine/CX3CL1 were significantly higher and the concentrations of SDF-1α/CXCL12 and CD30L/CD153 were significantly lower in M3.stop-infected mice. At day 7 p.i., the only significant difference in the concentrations of chemokines between the groups was a lower level of CD30L/CD153 in M3.stop-infected mice. Of note, also, the levels of KC/CXCL1 and BLC/CXCL13 did not vary significantly between M3.stop and M3.MR-infected mice. Thus, the difference in levels of these chemokines that was seen between the groups in the array experiment above was not substantiated.

Thus, our results are consistent with the notion that M3 functions primarily through its direct interactions with cellular chemokines, and that these interactions are critical for the efficient establishment of persistent infection in the wood mouse.

## Discussion

Here we have shown that the MHV-68 *M3* gene, which encodes a highly expressed chemokine-binding protein [15], [16], contributes substantially to infection in the lung and spleen of wood mice (*Apodemus sylvaticus*), which we have conclusively shown to be a natural host of MHV-68 [21]. This work was prompted by our finding that experimental MHV-68 infection of wood mice differs in several key respects from infection of BALB/c mice (*Mus musculus*) [22], and an earlier demonstration that, surprisingly, inactivation of the *M3* gene has little consequence in the context of comparable (intra-nasal) MHV-68 infection of inbred strains of laboratory mice [17], a now widely utilized small-animal model of γ-herpesvirus infection. Specifically, M3 contributes to the formation of iBALT, and the spike in latently infected cells within spleen that occurs at approximately 2 weeks p.i. and the level of long-term latency. While iBALT is not evident in the lungs of infected BALB/c mice [22], inactivation of M3 expression from the same mutant virus (M3.stop) did not have a comparable effect in CD1 and C57BL/6 mice on this transient rise in latently infected splenocytes [17], a feature common to infection in both strains. Thus, the contributions of M3 to MHV-68 infection are largely species specific, though an attenuation of MHV-68 infection in brain as a result of an altered inflammatory (predominantly neutrophilic) response has been observed in CD1 mice injected intracerebrally with M3.stop relative to M3.MR virus [17], suggesting that M3 is not fully inactive in *Mus musculus*.

In contrast to the lack of an attenuation of either lytic or latent MHV-68 infection previously observed in CD1 and C57BL/6 mice following intranasal inoculation with M3.stop virus [17], in an earlier report BALB/c mice infected via the same route with a virus in which the *M3* gene had been replaced with a *LacZ* expression cassette exhibited a reduced viral latent load in the spleen [33]. Further, depletion of CD8^+^ T cells partially precluded this effect, suggesting a role for M3 in the inactivation of chemokines involved in the T-cell response [33], which peaks between 10 and 20 days p.i. [6], i.e., the point at which the consequences of ablation of M3 expression were most evident in spleen. This phenotype, however, is similar to that observed in BALB/c and C57BL/6 mice in three independent reports of infection with MHV-68 *M2* mutants [35], [36], [37]. Because of this, and that the 5′ regulatory region of the *M2* gene extends into the adjacent M3 ORF [29], [38], we believe it is very likely that the apparent effects of disrupting *M3* expression by insertion of a CMV promoter-*LacZ* cassette in this earlier study may have been due instead to a combination of removing the *M3* ORF and/or an unintended disruption of M2 expression or an immune response to *LacZ* [39]. Since we also observed a reduction in latent virus load in the spleens of infected wood mice here as a consequence of specifically targeting M3 expression (Fig. 6), it will be interesting to determine if loss of M2 contributes also to this phenomenon within this host, as it does in *Mus musculus*.

In addition to species-associated differences seen within lungs upon MHV-68 infection, the spleens of infected wood mice exhibit clearly defined secondary follicles with highly organized germinal centers, whereas the follicles in BALB/c mice are notably larger and poorly organized [22]. Interestingly, follicles containing infected splenocytes in wood mice that had been inoculated with M3.stop virus (Fig. 5) appeared very similar morphologically to those that we observed in the spleens of BALB/c mice infected with MHV-68[22], indicating that this additional difference between mouse species may also be due to the presence or not of M3.

It was surprising, given that M3 modulates the action of a number of macrophage-specific chemokines that granulomatous infiltrations were present in similar number and size in both M3.stop and M3.MR-infected wood mice. These are most prominent at day 7 p.i., are macrophage rich and are the focus of MHV-68 replication in the lung [22]. This is perhaps due to the location of M3 expression, which is in B cells in perivascular/peribronchiolar infiltrates and iBALT but conspicuously not in granulomatous infiltrates. This suggests that the effect of M3 on chemokines is localized predominantly to areas where M3 is expressed.

Perhaps the most significant observation is that iBALT in the lungs of acutely infected wood mice is dependent on M3. iBALT is an example of tertiary or ectopic lymphoid tissue that develops at any sub-epithelial site in response to inflammation or infection. The organization of tertiary lymphoid tissue is remarkably similar to that of secondary lymphoid tissues with separate B and T cell areas, a network of specialized dendritic cells, and the presence of high endothelial venules [reviewed in [40]]. Additionally, their organization is dependent on the same chemokines that are required in lymph nodes [41]. Although the purpose of iBALT is not completely understood, it has been proposed that it participates in generation of protective immune responses along with secondary lymphoid tissue. For instance, in the absence of secondary lymphatic tissues (using *Lta^−/−^* mice), iBALT has been shown to provide protective immunity to influenza virus infection, as it is able to generate isotype-switched B cells via germinal center reactions and specific CD8^+^ T cells [42]. In contrast, in MHV-68 infection iBALT does not appear to play a protective role as in its absence productive infection is not greater, and in fact latency is attenuated. Instead, we hypothesize that MHV-68, via M3 functions, utilizes iBALT as a means to augment virus persistence by promoting B cell proliferation, as numerous latently infected cells can be found in these B cell-dominated accumulations (Fig. 3) that we have shown are devoid of viral structural antigens, and thus presumably virus replication, which occurs primarily within pulmonary granulomatous infiltrates in wood mice [22]. At the present time, the phenotype of the T cells (CD3^+^) present in the iBALT is not known, but it is plausible that these are either CD4^+^ T cells that would promote the activation of B cells by providing the necessary CD40, or a subset of CD8^+^ T cells (IFN-γ-secreting, CD40L^+^, perforin negative) that are necessary for ectopic lymphoid follicle formation [43].

Generation of iBALT and highly organized germinal centers are events that rely heavily on coordinated cell migration and organization, for which chemokines are critical. Given the chemokine-binding properties of M3 that have been demonstrated *in vitro* [15], [16], and the altered inflammatory response to MHV-68 infection in brain as a consequence of eliminating M3 expression [17], we asked whether these events associated with MHV-68 infection in wood mice reflect an M3-dependent change in the pulmonary chemokine/cytokine profile (Figs. 7, 8). Our array-based analysis of chemokine and cytokine levels revealed that numerous T cell, monocyte/macrophage, and neutrophil associated chemokines were present in higher levels within the lungs of wood mice infected with M3.stop relative to M3.MR. For example, levels of the chemokines RANTES/CCL5, MIP-1α/CCL3, MIP-1γ/CCL9, MIG/CXCL9, MIP-2/CXCL2, MIP-3β/CCL19 and fractalkine/CX3CL1 were lower in the wood mice infected with wild-type virus. In contrast, analysis revealed that the levels of two factors, SDF-1α/CXCL12 and CD30L/CD153 were higher after infection with M3.MR in both the array and ELISA assays (Figs. 7, 8). With respect specifically to iBALT formation, stromal chemokines such as SDF-1α/CXCL12 (levels enhanced by M3) have been implicated in the cellular recruitment required for iBALT formation [44]. Additionally, CD30L has a role in the segregation of B and T cells within the murine spleen [45] and so may have an as yet uncharacterized role in iBALT formation. MIP-3β/CCL19 is involved in lymphocyte recruitment, and inhibition by M3 has been proposed as a survival advantage for MHV-68 [46]. Our observations are in agreement with this hypothesis. Moreover, BALT is spontaneously-produced in mice that are deficient in the receptor for CCL19 (CCR7^−/−^), a phenomenon that is related to a defect in homing of regulatory T cells [47]. In contrast, MIP-3β/CCL19 has been implicated in iBALT formation in lymph-node and spleen-deficient laboratory mice [41], which is at odds with our results. Thus, iBALT formation is complex and modulation in the levels of factors such as SDF-1α/CXCL12, CD30L/CD153 and MIP-3β/CCL19 by M3 may contribute to the formation of iBALT in context of MHV-68 infection in wood mice.

As noted above, a number of T cell, monocyte/macrophage, and neutrophil associated chemokines were present in higher levels within the lungs of wood mice infected with M3.stop relative to M3.MR. RANTES/CCL5 is an important proinflammatory chemokine that induces the recruitment of T cells (including CTLs), monocytes and eosinophils to the sites of virus infection. Other studies have shown that blocking RANTES/CCL5 *in vivo* significantly increases the titers of respiratory syncytial virus in the lungs of infected mice, and this is associated with reduced T cell recruitment [48] and heightened lung disease. Additionally, influenza virus infection of MIP-1α/CCL3^−/−^ mice leads to a reduced inflammatory response and increased virus titers [49]. MIP-2/CXCL2 induces neutrophil recruitment [50]. Hence, inhibition of such chemokines by M3 conceivably favors MHV-68, not necessarily to increase virus replication, but to promote establishment of latent infection and virus persistence, a hallmark property of all herpesviruses.

Leptin receptor and its ligand (an IL-6 family member) were expressed at elevated levels in the lungs of M3.stop infected wood mice (22-fold and 10-fold respectively). Leptin is an adipocyte-derived cytokine that regulates energy intake and expenditure. However, leptin promotes Th1 immune responses as well as inducing cytokine secretion and increasing phagocytosis by macrophages (reviewed in [51]). Deficiency in leptin production has also been associated with susceptibility to pulmonary disease in a mouse model [52]. Thus, modulation of leptin by the indirect action of M3 may confer a survival advantage for MHV-68.

The cellular and biochemical consequences of M3 expression are clearly complex. M3 is a chemokine-binding protein, and as such is thought to disrupt chemokine gradients, modulating the response of cells *in vivo*. Thus, a lack of M3 should increase recruitment of cells that respond to the chemokines bound by M3. Thus, the changes in cytokine and chemokine profiles that we observe may be due to modulation of the composition of infiltrating cell types and the activation status of these cells. Nonetheless, levels of the chemokines RANTES/CCL5, MIP-1α/CCL3, MIP-3β/CCL19 and fractalkine/CX3CL1 that are known to be bound or functionally impaired by M3 [15], [16], [46] were lower in the wood mice infected with wild-type virus, and thus a direct effect of M3 on chemokine levels could also play a role.

At this juncture, it is unclear what the basis is for the lack of an apparent influence of M3 in the context of MHV-68 intra-nasal infection in laboratory strains of mice [17]. Given the relatively close genetic relationship between *M. musculus* and *A. sylvaticus*, that there is such a notable difference in the role of M3 is surprising, particularly since there is a change in the inflammatory response (predominantly neutrophilic) within brain to MHV-68 infection following intracerebral inoculation of CD1 mice with M3.stop virus [17]. This response is distinct from that seen after intranasal infection of wood mice where few neutrophils are present, but suggests that M3 is indeed capable of functioning within *M. musculus*, and that the absence of an apparent influence of M3 in the lung and spleen in this host, therefore, may be due to relatively subtle differences between this species and the natural host. One possibility is that M3 expression in the lung and spleen of a *M. musculus* host is below a critical threshold. However, comparative analyses of MHV-68 mRNA expression in *M. musculus*-derived cells, albeit within infected cells *in vitro*, have revealed that *M3* is one of the most highly expressed MHV-68 genes during the virus lytic cycle [27]. When we assessed *M3* mRNA levels in the lung of infected wood mice and BALB/c mice at 7 and 14 days p.i., *M3* expression in BALB/c mice was similar to wood mice at day 7, but significantly lower at day 14 (Fig. 1B). Our observed kinetics of M3 expression in BALB/c mice fits with a previous study [53]. Additionally, high levels of *M3* mRNA were detected much later (14 days p.i.) in wood mouse lungs than mRNAs from the other genes in this locus (Fig. 1A), suggesting that the timing of *M3* expression may be important, and that in *M. musculus* the lower level (approx. 10 fold) of *M3* at day 14 p.i. may be critical. In this context, previous work has shown that the peak of chemokine expression in MHV-68–infected BALB/c mice occurs after the peak of M3 expression [53], [54] and that deletion of the *M3* locus does not affect chemokine levels [53]. Alternatively, the cellular source and location of M3 may play a role. While this may reflect latency-associated *M3* expression, at this time we also detected equivalent levels of mRNA from *ORF50*, a key gene of the lytic cycle. Finally, a possibility worthy of consideration is that minor species differences in cytokine(s) protein structure have combined with coding changes in *M3* that have occurred during passage of MHV-68 *in vitro* to render M3 less effective within *M. musculus.* Such changes in M3 would be possible due to a reduction in selective pressure to retain *M3* integrity *in vitro*, where it neither contributes directly to nor is it essential for MHV-68 replication [17], [33].

In summary, the results from this study demonstrate that M3 is important for MHV-68 infection by facilitating an environment in which proliferating B cells would accumulate, both during iBALT formation in the lungs and the germinal center reaction in the spleen. These responses ultimately lead to efficient establishment and augmentation of MHV-68 latency, in both the lungs and spleens of its natural host. Significantly, this work also highlights the importance of using the natural host for studying the role of virus genes, particularly those involved in modulating the innate and adaptive host antiviral response, whose functions have no doubt intricately evolved within the context of a specific host.

## Materials and Methods

### Ethics statement

All animal work was performed under strict accordance with UK Home Office guidelines and approved by the UK Home Office under Project Licence numbers 40/2483 and 40/3403 and Personal Licence number 60/6501.

### Mice

Wood mice (*Apodemus sylvaticus*) were obtained from an out-bred colony established at the University of Liverpool, Faculty of Veterinary Science [55], [56]. This colony was obtained from Dr. J. Clarke in 1995, and derived from captive-bred colonies maintained for several decades in the Department of Zoology, University of Oxford, UK with only occasional introductions of new stock from the wild. Their general housing and maintenance has been described elsewhere [57], and at Liverpool they are maintained under semi-barrier conditions. The Liverpool colony has suffered no clinical disease, and, although not specified pathogen free (SPF) in the sense used for most laboratory rodents, samples are tested routinely on a monthly basis for the major infections of laboratory rodents have so far been negative. Of particular relevance to this study, no evidence of MHV-68 infection has been found in the colony by serology and PCR analysis [19]. Animals were anesthetized with isoflurane and inoculated with 4×10^5^ plaque forming units (PFU) in 40 µl of sterile phosphate buffered saline (PBS). At various times between day 3 and 40 p.i., animals were euthanized and tissues were harvested.

### Cell culture and virus

Stocks of MHV-68, clone g2.4 [58], and previously published mutant MHV-68 viruses M3.stop and M3.MR [16] were grown and titrated by infection of baby hamster kidney cells (BHK-21), as previously described [23]. BHK-21 cells were maintained in Glasgow's Modified Minimal Essential Medium with 10% newborn calf serum and 10% tryptose-phosphate broth, 2 mM L-glutamine, 70 µg/ml penicillin and 10 µg/ml streptomycin. NIH3T3 cells were maintained in Dulbecco's Modified Eagles Medium (DMEM) supplemented with 10% fetal bovine serum, 2 mM L-glutamine, 70 µg/ml penicillin and 10 µg/ml streptomycin.

### Quantitative Reverse Transcription PCR (qRT-PCR)

Total RNA was purified from lung tissue using the RNeasy Mini Kit (Qiagen) and DNA contamination removed by treating RNA with amplification grade DNase I (Invitrogen) according to the manufacturers' recommendations. Reverse transcription was performed at 50 °C for 30 min with 2 µg RNA in a 20-µl reaction containing 200 U Superscript III reverse transcriptase (Invitrogen), 500 ng oligo(dT)_15_ primer (Roche), 0.5 mM dNTP mix (Promega), 5 mM DTT, 40 U RNase inhibitor (RNaseOUT; Invitrogen), and First-Strand buffer (50 mM Tris-HCl [pH 8.3], 75 mM KCl, 3 mM MgCl_2_; Invitrogen). Afterwards, 2 µl was used as template for qRT-PCR in 20-µl reaction volumes. Quantification of cDNA was done using an Opticon Monitor 2 real-time PCR machine (MJ Research) with DyNamo SYBR Green kit (Finnzymes) and 0.5 µM of each oligodeoxynucleotide primer (the oligodeoxynucleotide primers used for PCR and qRT- PCR are provided in Table 1). The cycling parameters were initially 95 °C for 10 min, and then for each cycle: 94 °C for 10 s, 60 °C for 20 s, and 72 °C for 15 s. Melting curve analysis was carried out between 65–95 °C with 0.2 °C increments to confirm product specificity. For each individual experiment, amplification of cDNA from the murine ribosomal protein L8 mRNA (*RPL8*; accession # AF091511) was used to normalize for input cDNA between samples using exon-spanning primers to control for contaminating cellular DNA. Each sample was amplified in triplicate, and mean cDNA copy numbers were determined from three individual mice and expressed relative to the copy number of *RPL8* cDNA.

**Table 1:** Oligodeoxynucleotide primers used.

Target	Primer Sequence (5′-3′)	Product length	Notes
MHV-68 *M1*	GACTGCCCTTGTCACTTTTC	126 bp	qRT-PCR
	CCAGGTAAGAGATCCTGTGT		
MHV-68 *M2*	GACAGTCCAGAAAATCTAGGC	110 bp	qRT-PCR
	ATGACATTTGGATGGTGGAATA		
MHV-68 *M3*	CCCCATCATGACTTGTCATC	205 bp	qRT-PCR
	AAAACTTGCCCATGCTACT		
MHV-68 *M4*	TTTTCGATCAGCCACGGTTG	139 bp	qRT-PCR
	CATCGACACAACGGATTTGATA		
MHV-68	ACCAGAAGGTGAGGTTTAATGC	175 bp	qRT-PCR
*ORF50*	GAAGTGCGAGCTGTGGGTT		
Mouse	CAGTGAATATCGGCAATGTTTTG	163 bp	Normalize vDNA copies
Genomic *RPL8*	TTCACTCGAGTCTTCTTGGTCTC		
MHV-68 *M3*	CTCTGGGAGAGCGTCAG	1248 bp	Product ligated into pCRII
	GTTACTGAGTATCAATGATCC		to generate RNA-ISH probes
Mouse *RPL8*	ACAGAGCCGTTGTTGGTGTTGT	100 bp	*RPL8* (exon spanning)
mRNA	CAGTTCCTCTTTGCCTTGTACT		used to normalize cDNA copies
MHV-68	CTACTTCTTCATCGGACGCT	159 bp	MHV-68 *gp150*
*gp150*	CGGGATCTGTCGGACTGT		quantification of MHV-68 vDNA

### Virological analyses

Quantification of viral DNA copy number (per 200 ng DNA) was determined as previously described [59] using PCR primers specific for the MHV-68 *gp150* gene. The *RPL8* gene was used to normalize for input DNA between samples. Mean viral genome copy numbers were determined from three or four individual infected animals. Splenocytes isolated from intact spleens were examined for latent virus by an infective center assay using NIH3T3 cells, as previously described [23].

### Histology, immunohistology and *in situ* hybridization

Lung, spleen, and lymph node tissue were fixed in 4% buffered paraformaldehyde and routinely embedded into paraffin wax. Sections (3–5 µm) were either stained with haematoxylin and eosin, or used for immunohistology or RNA *in situ* hybridization. Immunohistology was performed using the peroxidase anti- peroxidase and the avidin biotin peroxidase complex method as previously described [60]. T cells were detected using rabbit anti-human CD3 antibody (DAKO Cytomation). B cells were identified using rat anti-mouse CD45R (clone RA3-6B2; SouthernBiotech). Quantification of B and T cells was performed by counting the number of cells in corresponding sequential sections identified by the above antibodies. Five randomised areas of interstitial and perivascular/peribronchiolar infiltration and all areas of iBALT in the lung sections were analysed, using images captured with Nikon NIS-Elements Basic Research v3.0 software at 20× magnification. The proportion of B and T cells was then calculated for each area of peribronchiolar/perivascular infiltration and iBALT, and the number of cells per unit area for the interstitial infiltrate. These data are shown as the mean values±SEM and compared between groups using a two sample *t*-test. Detection of MHV-68 *M3* RNA and *vtRNAs* by RNA *in situ* hybridization followed a previously described protocol [61]. Briefly, digoxigenin (DIG)-labeled sense and antisense probes were generated by *in vitro* transcription, using the DIG RNA labeling kit (Roche), of either the entire *M3* ORF that was amplified from MHV-68 DNA (see Table I for primers used) and cloned into pCRII (Invitrogen) or transcripts to the MHV-68 *tRNA* genes 1-4 within plasmid pEH1.4 as described previously [10]. Briefly, sections were treated with proteinase K (1 µg/ml; Roche) at 37 °C for 15 min, and hybridization performed overnight at 52 °C. Hybridized probe was detected with alkaline phosphatase-conjugated anti-DIG Fab fragments (Roche) and BCIP/NBT (Sigma). Slides were counterstained with Papanicolaoùs hematoxylin.

### Chemokine/cytokine array analysis

Lungs were screened for expression of 61 cytokines/chemokines using a RayBio Mouse Cytokine Antibody Array Kit (Array 3.1.; Ray Biotech Inc., Norcross, GA), performed according to the manufacturer's instructions. Lung tissue (20–30 mg) taken from wood mice 14 days p.i. with either M3.stop or M3.MR was homogenized in 500 µl lysis buffer (RayBiotech) containing 1% (w/v) sodium deoxycholate, 2% (v/v) NP-40, 0.2% (w/v) SDS, 1 µg/ml each of aprotinin, leupeptin, pepstatin, and 1 mM phenylmethylsulfonyl fluride (PMSF) on ice. Protein concentrations were determined using a BioRad *DC*-Protein Assay Kit according to the manufacturer's instructions. As an extra control, 25 µg protein from each sample was analyzed by western blot to detect actin to ensure analysis of equal starting material (data not shown). Cell lysates were sent to RayBiotech (RayBiotech, Inc. 3607 Parkway Lane, Suite 200, Norcross GA 30092, U.S.A.) for analysis of chemokine and cytokine levels using the RayBio Mouse Cytokine Antibody Array 3.1 kit (RayBiotech), using 500 µg protein per membrane. Signals were detected and quantified by chemiluminescence.

### Chemokine concentration analysis

Lungs were screened for expression of specific chemokines by ELISA. Lung tissue (20–30 mg) taken from mice was homogenized in 1 ml of ice-cold T**-**PER Tissue Protein Extraction Reagent (Pierce) in the presence of protease inhibitor cocktail (Sigma-Aldrich) before being clarified by centrifugation (10,000 *g* for 5 minutes at 4 °C). Total protein concentrations were determined by using *DC*-Protein Assay Kit (BioRad) according to the manufacturer's instructions. Chemokine concentrations were measured using DuoSet ELISA Development systems for RANTES/CCL5 (DY478), KC/CXCL1 (DY453), fractalkine/CX_3_CL1 (DY472), SDF-1α/CXCL12 (DY460), BLC/CXCL13 (DY470) and CD30 Ligand (CD153) (DY732) in accordance with manufacturer's instructions (R&D Systems Europe Ltd., Abingdon, UK). Lung tissues lysates were investigated in duplicate and diluted as appropriate to ensure protein concentrations were within the linear range of the standard curve. Optical densities were determined at 450 nm using a Thermo Labsystems Opsys MR ELISA plate reader (Thermo Life Sciences, Basingstoke, UK).
